# Structural insight into *Vibrio cholerae* EIIC sugar transporter dimer captured in a substrate-free inward-facing state: *Vibrio cholerae* EIIC dimer in inward-facing state

**DOI:** 10.3724/abbs.2025120

**Published:** 2025-08-12

**Authors:** Hanhan Guo, Qiaoshuo Zhang, Zhao Wang, Kuo Zhang, Yang Fu

**Affiliations:** 1 Department of Biochemistry SUSTech Homeostatic Medicine Institute School of Medicine Southern University of Science and Technology Shenzhen 518055 China; 2 Institute for Biological Electron Microscopy Southern University of Science and Technology Shenzhen 518055 China

**Keywords:** PTS system, EIIC transporter, cryo-EM

## Abstract

The phosphoenolpyruvate-dependent sugar phosphotransferase system (PTS) is a central pathway for carbohydrate transport in bacteria and plays a critical role in nutrient acquisition, metabolism, and virulence. In *Vibrio cholerae*, the glucose-specific EIIC transporter is a key component of the PTS system, mediating the transport of sugars into the bacterial cell, coupled with phosphorylation during translocation. Here, we present the 3.68 Å cryo-electron microscopy (cryo-EM) structure of the dimeric EIIC transporter from *Vibrio cholerae* in its inward-facing, substrate-free conformation. The structure reveals a detailed arrangement of the scaffold and transport domains, stabilized by extensive inter- and intraprotomer interactions. Comparative analysis with substrate-bound inward-facing structures of EIIC from *E*. *coli* highlights conformational changes, providing insights into substrate release and the structural transitions required for alternating access. Notably, the observed substrate-free inward-facing conformation features a larger substrate-binding pocket, which is consistent with a state poised for glucose release into the cytoplasm. The formation of a unique intraprotomer disulfide bond between residues C240 and C254 stabilizes the interface between the scaffold and transport domains, potentially regulating transporter dynamics. These findings elucidate the structural basis for substrate release in the PTS system and underscore the dynamic nature of EIIC-mediated sugar transport. Our study enhances the understanding of PTS system function in *Vibrio cholerae* and highlights the EIIC transporter as a promising target for antimicrobial drug development. Disruption of sugar transport in this essential pathway could impair bacterial growth and virulence, suggesting a novel therapeutic strategy against cholera. These results provide a foundation for future investigations into the structural and functional dynamics of bacterial sugar transporters.

## Introduction

The phosphoenolpyruvate-dependent sugar phosphotransferase system (PEP-PTS) is a fundamental mechanism by which bacteria acquire and metabolize sugars. This system utilizes phosphoenolpyruvate (PEP) to transfer a phosphate group to sugars during their transport across the bacterial membrane [ 1– 3] . The PTS system is not only for nutrient acquisition and metabolism but also for bacterial virulence and adaptability to changing environmental conditions [ 4– 12] . In *Vibrio cholerae*, the causative agent of cholera, the PTS system is indispensable for its ability to colonize diverse niches, including the human intestine. Efficient acquisition and metabolism of sugars such as glucose, mannose, and N-acetylglucosamine (NAG) are vital for bacterial survival, energy production, and virulence factor expression [ 13– 17] .

The PTS system comprises three main components: enzyme I (EI), heat-stable phosphocarrier protein (HPr), and enzyme II (EII). EI and HPr function as sugar-nonspecific energy-coupling proteins, whereas EII facilitates substrate-specific transport. The EII complex consists of EIIA, EIIB, and the membrane-integral EIIC, which mediate sugar transport via conformationally driven alternating access [ 3, 18] . Structural insights into EIIC are essential for understanding substrate recognition, transport specificity, and conformational transitions during sugar translocation. The inward-facing conformation, in which the transporter is poised to release sugar molecules into the cytoplasm after translocation, represents a crucial stage in the transport cycle. High-resolution structural data of this conformation can illuminate the molecular mechanisms underpinning substrate release and transporter dynamics [19].

Inhibiting the EIIC sugar transporter in *Vibrio cholerae* impairs key systems that the bacterium uses to sense and respond to nutrient availability, leading to several downstream effects that reduce its pathogenic potential. Specifically, blocking EIIC impairs sugar uptake and metabolism, disrupting central metabolic pathways essential for energy production and growth in nutrient-variable environments such as the intestine [20]. Furthermore, the loss of EIIC function significantly reduces biofilm formation by impairing necessary signaling pathways, thereby affecting environmental persistence and early host colonization stages [21]. Additionally, disrupted EIIC-mediated sugar transport interferes with nutrient-sensing circuits regulating key virulence factors, including cholera toxin, undermining the ability of bacteria to establish infection [22]. Thus, inhibiting EIIC in *Vibrio cholerae* profoundly affects sugar uptake, biofilm formation, and virulence gene activation, collectively undermining bacterial survival and pathogenicity.

Given the critical role of EIIC in sugar uptake and its involvement in the metabolic pathways that support *Vibrio cholerae* virulence, the transporter presents an attractive target for antimicrobial drug development. Disruption of EIIC-mediated sugar transport could impair bacterial growth and pathogenicity, suggesting a novel therapeutic approach. Cryo-electron microscopy (cryo-EM), a powerful tool for resolving membrane protein structures, enables detailed analysis of EIIC of *Vibrio cholerae*, offering insights into bacterial pathogenesis and therapeutic opportunities for combating cholera and related infections [23].

In this study, we present the high-resolution cryo-EM structure of the EIIC transporter from *Vibrio cholerae*, revealing its inward-facing, substrate-free conformation. The structure provides a detailed view of the dimeric architecture, including the scaffold domain (SD) and transport domain (TD), as well as the extensive interdomain and interchain interactions that stabilize the assembly. The observed conformation, likely representing the substrate-released state, highlights key features involved in substrate translocation and release. Comparative analysis with substrate-bound inward-facing structures revealed significant conformational changes in critical regions, such as the amphipathic helix (AH) and associated helical hairpins (HP1 and HP2), which facilitate the dynamic transitions required for sugar transport. These structural insights contribute to our understanding of the transporter’s function in the PTS system of *Vibrio cholerae*, laying the groundwork for future studies on its dynamic mechanisms and potential therapeutic targeting.

## Materials and Methods

### Protein expression and purification

The gene encoding the EIICB protein from *Vibrio cholerae* was subsequently cloned and inserted into the expression vector pET-21a with a Strep tag at the C-terminus. *E*. *coli* BL21 (DE3) cells harboring the expression plasmid were grown in LB media supplemented with ampicillin at 37°C. Protein expression was induced via the addition of 0.5 mM IPTG at 16°C for 20 h when the culture reached an OD600 of 0.6. The cells were collected by centrifugation at 4000 *g* for 20 min and lysed in lysis buffer (30 mM HEPES, pH 7.5, and 150 mM NaCl). The lysate was centrifuged at 12,000 *g* at 4°C for 30 min to remove unbroken cells, and the supernatant was further centrifuged at 100,000 *g* at 4°C for 60 min to collect the membrane. The membrane pellet was resuspended and incubated in lysis buffer containing 1% N-dodecyl-β-D-maltoside (DDM) at 4°C for 2 h and centrifuged at 20,000 *g* at 4°C for 30 min to remove insoluble material. The supernatant was applied to Strep-Tactin® Sepharose resin (IBA Lifesciences, Goettingen, Germany). The column was washed with 20 column volumes of wash buffer (30 mM HEPES, pH 7.5, 150 mM NaCl, and 0.01% LMNG), and the recombinant protein was eluted via wash buffer supplemented with 10 mM D-biotin (IBA Lifesciences, Goettingen, Germany). The eluted fractions were further purified through a Superdex 6 increase 10 / 300 GL column (Cytiva, Marlborough, USA).

### Cryo-EM sample preparation and cryo-EM data collection

For cryo-EM sample preparation, a small aliquot of purified EIIC protein in LMNG micelles was applied to a glow-discharged Quantifoil R1.2/1.3 300 mesh copper grid. The grids were blotted for 3–4 s using a Vitrobot Mark IV (Thermo Fisher Scientific, Waltham, USA) at 4°C and 100% humidity before being rapidly frozen in liquid ethane. Data were collected on a Titan Krios microscope (Thermo Fisher Scientific, Waltham, USA equipped with a K3 Summit direct electron detector (Gatan, Pleasanton, USA) operating in counting mode. The microscope was operated at an accelerating voltage of 300 kV with a nominal magnification of 130,000×, resulting in a pixel size of 0.668 Å. A total of 5864 movies were collected over a defocus range of –1.5 to –2.5 μm, with a total dose of 50 e ^–^/Å².

### Cryo-EM data processing

All the cryo-EM data were processed via the cryoSPARC software suite (version 3.2). The initial dataset was processed by first performing motion correction on the collected movies via the “Patch Motion Correction” algorithm, followed by contrast transfer function (CTF) estimation with the “CTF Refinement” module. Particles were automatically picked from the micrographs via the “Blob Picker” method, and a subset of picked particles was subjected to 2D classification to identify the best particle orientations and to remove low-quality particles.

A total of 305,498 particles were initially selected for three-dimensional (3D) classification. After 3D refinement of the initial model, a set of high-quality particles was chosen, and 3D classification with a reference model was performed to refine the structural heterogeneity. The final 3D reconstruction was obtained with a global resolution of 3.68 Å on the basis of the gold-standard Fourier shell correlation (FSC) at the 0.143 threshold. All map sharpening and postprocessing were performed in cryoSPARC to obtain the final density maps.

### Model building and structural refinement

The initial model for the EIIC transporter was built via the cryo-EM density map obtained from the final 3D reconstruction. Manual model building and adjustment were performed in Coot to fit the cryo-EM density [ 24, 25] . The model was further refined via Phenix, which employs real-space refinement and geometrical restraints to minimize steric clashes and optimize bond lengths and angles. During refinement, the cryo-EM map was used to guide the placement of side-chain densities, and the refinement was iterated until the model converged to a satisfactory fit with the experimental data. The final model was evaluated for quality via the MolProbity server, which revealed favorable geometry with no steric clashes. The data collection, reconstruction, and model refinement statistics are summarized in Supplementary Table S1.

### Circular dichroism

Thermal stability experiments were carried out via circular dichroism (CD) on a J-810 spectropolarimeter (Jasco, Tokyo, Japan) equipped with a Peltier system for temperature control. All measurements of the EIIC samples were performed in 150 mM sodium chloride, 30 mM HEPES, pH 7.5, 150 mM NaCl, and 0.01% LMNG at a protein concentration of 0.1 mg/mL. The temperature ramp measurements were recorded from 30 to 98°C (temperature slope 2.0°C/min) in a 0.1-cm path length cuvette and monitored at 222 nm.

## Results

### Structure determination of the EIIC protein of *Vibrio cholerae*

**Figure 1:**
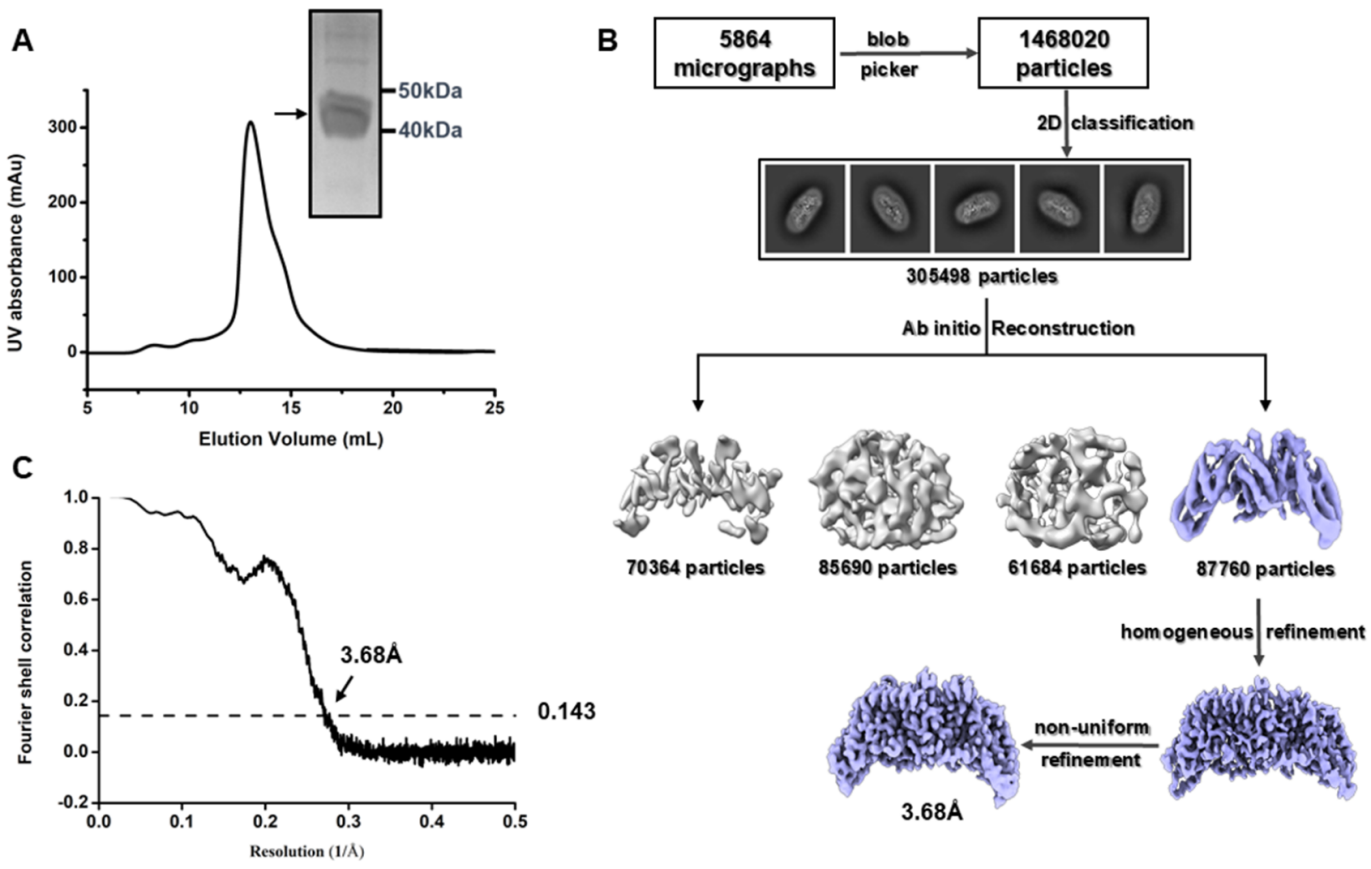
Figure 1 Purification and characterization of the EIIC transporter from *Vibrio cholera* (A) Size-exclusion chromatography (SEC) elution profile of EIIC on a Superose 200 column following Strep-Tactin affinity purification. Coomassie Brilliant Blue (CBB) staining of the SEC elution fractions confirmed the purity of the EIIC. (B) Cryo-electron microscopy (cryo-EM) workflow for EIIC. (C) Fourier shell correlation (FSC) curve estimating the final resolution of the EIIC dimer, which is based on the gold-standard FSC at the 0.143 threshold.

To elucidate the structural basis of carbohydrate transport by the glucose-specific PTS transporter in *Vibrio cholerae*, the EIIC protein was expressed in *E*. *coli*, embedded in lauryl maltose neopentyl glycol (LMNG), and analyzed via cryo-EM single-particle analysis (SPA) ( Figure 1A). A total of 5864 micrograph movies were collected on the Titan Krios TFS equipped with the K3 direct electron detector and processed with cryoSPARC software, including motion correction, contrast transfer function (CTF) estimation, and particle picking. After sorting the particles in 2D, data processing revealed the characteristic 2D class averages of the dimeric form of EIIC, with no density attributable to EIIB, indicating a lack of structural association between the two domains. After 3D classification and refinement, the best model (containing 87,760 particles) reached 3.68 Å resolution ( Figure 1B,C and Supplementary Figure S1).

### Overall structure of the dimeric EIIC transporter of *Vibrio cholerae*

**Figure 2:**
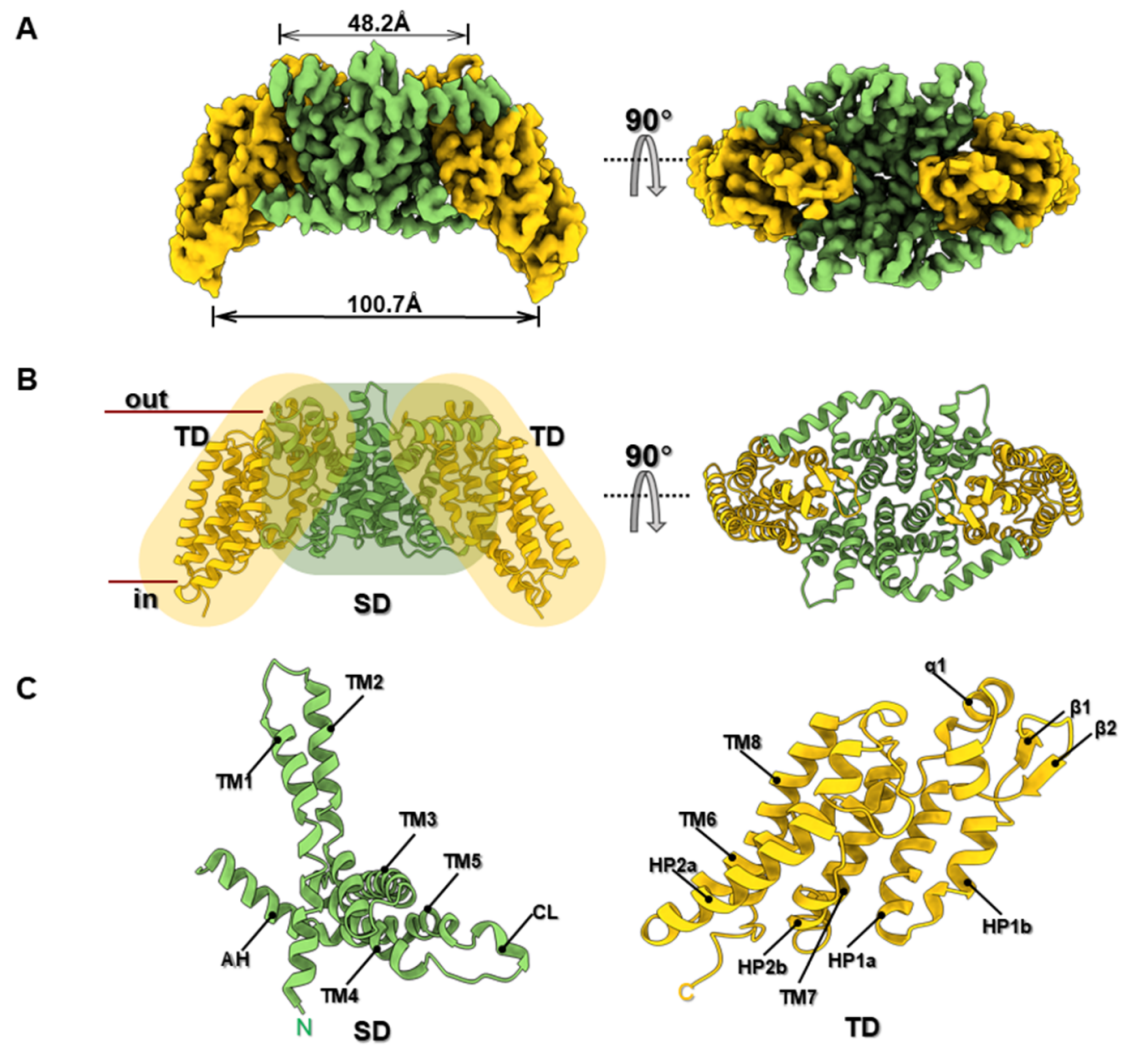
Figure 2 Structural overview of the EIIC from *Vibrio cholera* (A) Cryoelectron microscopy (cryo-EM) density map of the symmetric inward-facing EIIC dimer, highlighting its overall architecture. (B) Atomic model of the EIIC transporter shown from two perspectives: parallel to the membrane plane (side view) and an extracellular view (top view). The lipid bilayer interface is denoted by horizontal lines. The scaffold domains (SDs) and transport domains (TDs) are colored with different shades of green and orange, respectively. (C) Cartoon representation of the EIIC protomer. Key secondary structural elements are depicted.

The EIIC protein adopts a dimeric architecture, with two protomers aligned parallel within the membrane. The structure is reminiscent of an overturned canoe, characterized by a 100.7 Å wide concave surface oriented toward the cytoplasm and a narrower 48.2 Å wide surface facing the extracellular side ( Figure 2A). The protein is embedded within the detergent micelle, which mimics the membrane environment and facilitates high-resolution structural determination through cryo-electron microscopy. Each EIIC protomer consists of eight transmembrane (TM) helices, which are tightly packed in the membrane and exhibit high-resolution density, enabling precise identification of the membrane-spanning regions ( Supplementary Figure S1C). Notably, the structure adopts an inward-facing conformation, which is consistent with the previously resolved EIIC transporters from *B*. *cereus* and *E*. *coli* ( Supplementary Figure S2)[ 26, 27] .

**Figure 3:**
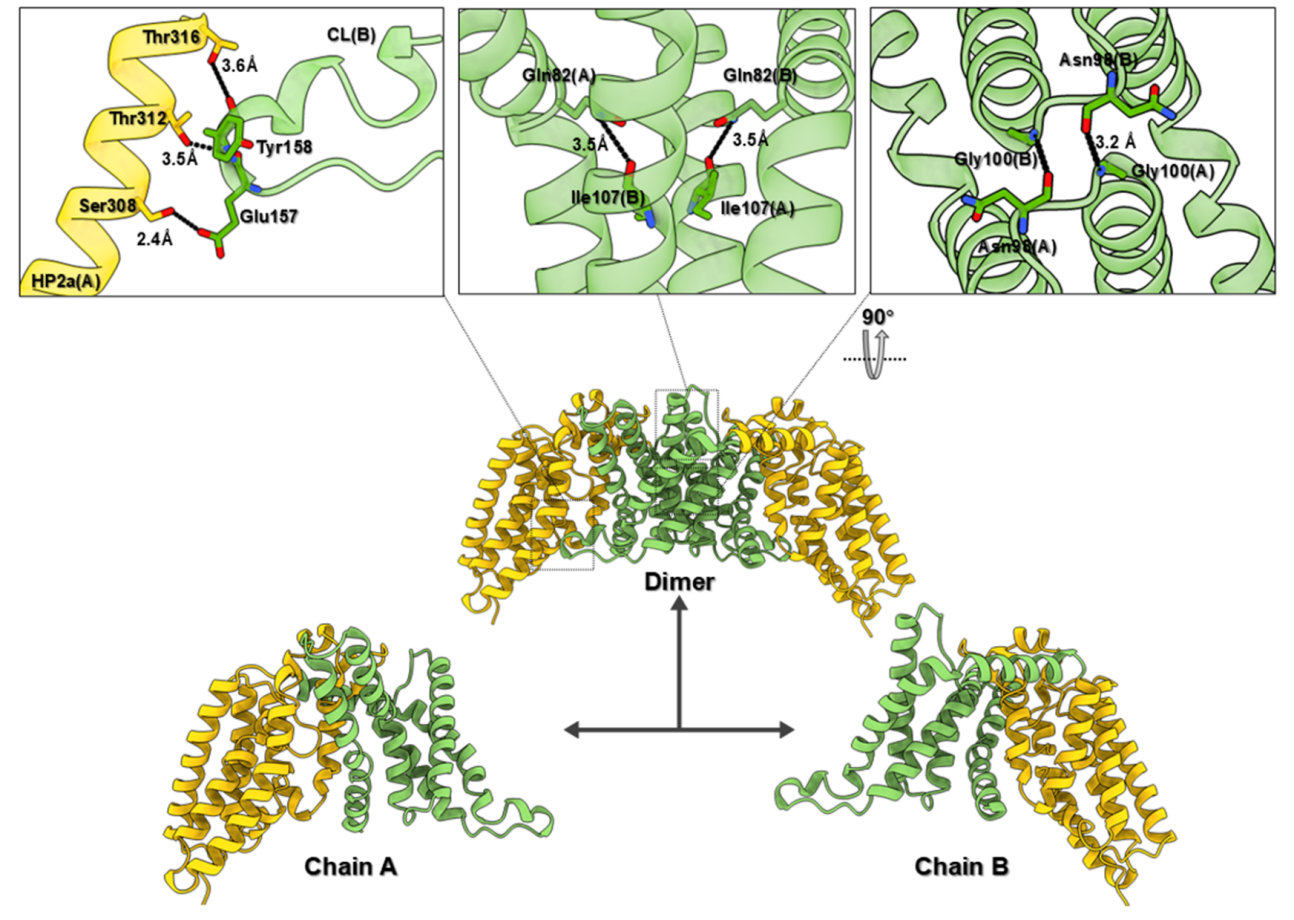
Figure 3 The dimerization interface of the EIIC from *Vibrio cholera* Cartoons of scaffold domains (SDs) and transport domains (TDs) are colored in green and orange, respectively. Key residues are shown as sticks. Dotted lines represent hydrogen bonds.

The protomers are organized into two functional domains: a scaffold domain (SD) at the N-terminus, which spans TM1–TM5, and a transport domain (TD) at the C-terminus, which spans TM6–TM8 ( Figure 2B,C and Supplementary Figure S3A). The SDs of the two protomers form a symmetric dimerization interface, which is predominantly stabilized by hydrophobic interactions between TM2 and TM3 of each protomer ( Supplementary Figure S3B). In addition to the hydrophobic contacts, interchain hydrogen bonds involving Q82-I107 and G100-N98 further contribute to the stability of the dimerization interface ( Figure 3). The TDs, located at the C-terminal ends of the protomers, are responsible for substrate binding and translocation. The amphipathic helix (AH) serves as a linker between the SD and TD, contributing to the overall structural integrity of the transporter. In addition to the AH, the protomer contains two α-helical hairpins (HP1 and HP2) that contribute to structural organization. HP1 is located immediately following AH, whereas HP2 is situated between TM6 and TM7 ( Supplementary Figure S3A). The first prominent periplasmic loop, situated between HP1 and TM6, consists of a β-hairpin followed by a short α-helix (α1), which likely interacts with the periplasmic space and stabilizes the transporter within the membrane environment (Figures 2 and 3).

A key feature of the dimeric EIIC transporter is the interdomain interaction between the SD and TD of adjacent protomers. The cytoplasmic loop (CL), located between TM4 and TM5, adopts an α-helix-like conformation and extends toward the TD of the neighboring protomer. Stabilization of this interchain interaction is achieved through hydrogen bonds between residues S308 and E157, T312 and E157, and T316 and Y158 ( Figure 3). The dimerization interface between the SD domains of the protomers involves an extensive surface area of approximately 2300 Å². This large interface is predominantly formed by hydrophobic interactions, with additional polar contacts providing structural stability. Residues S343 and T351, which form hydrogen bonds in the inward-facing state, are conserved across glucose-specific EIIC subfamily members, such as MalT from *B*. *cereus* and EIIC from *E*. *coli* ( Supplementary Figure S4). These conserved interactions highlight the evolutionary conservation of substrate translocation mechanisms within the PTS.

### Substrate-binding site of the EIIC transporter of *Vibrio cholerae*

All glucose (Glc) family EIIC transporters possess an almost universally conserved glutamate residue within the GITEP motif, which is essential for substrate transport and phosphorylation [ 3, 28] . Consistently, the EIIC transporter of *Vibrio cholerae* contains a conserved GITEP sequence, suggesting a preserved mechanism for substrate recognition across the Glc family ( Supplementary Figure S3C,D).

**Figure 4:**
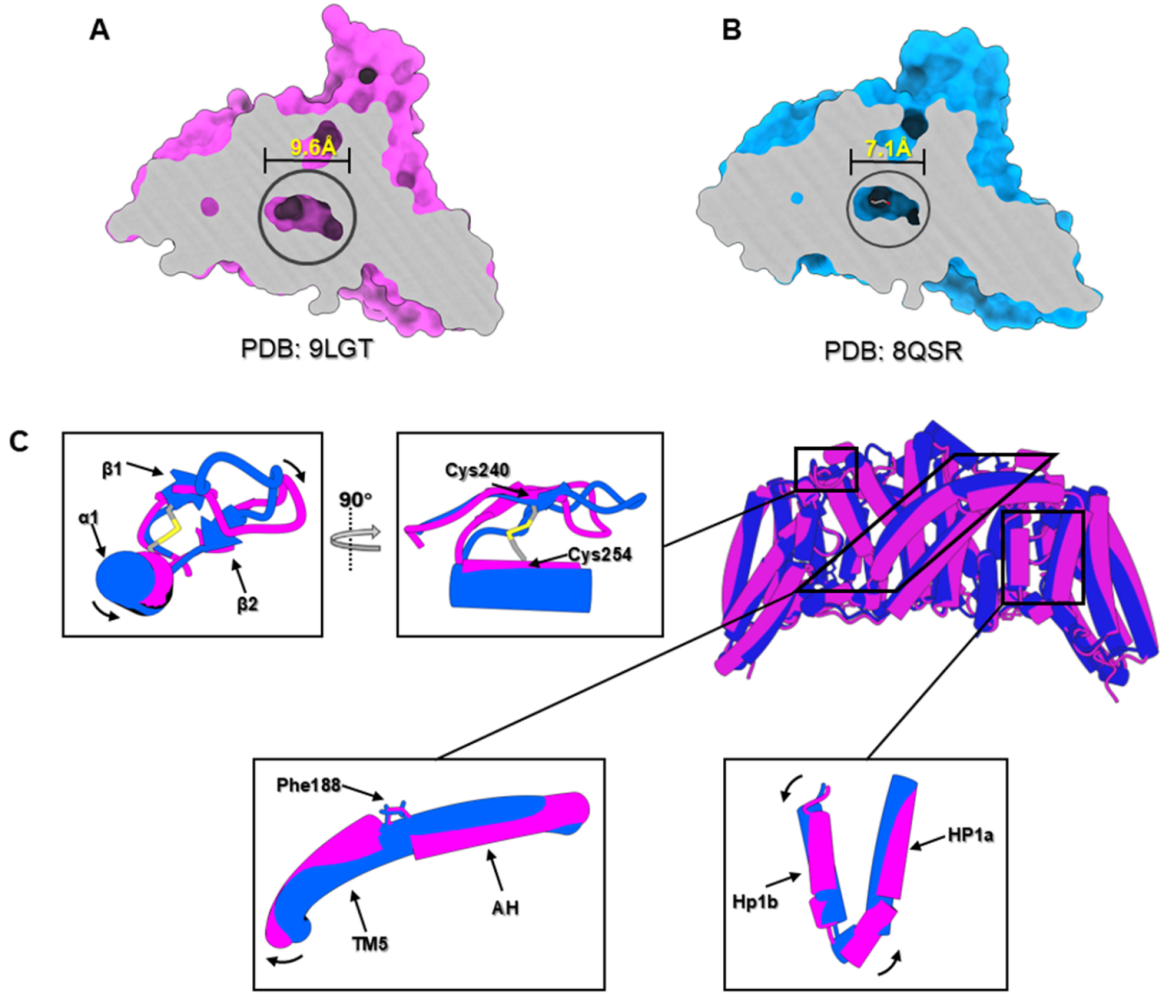
Figure 4 Structural features of the substrate-binding pocket in the inward-facing EIIC transporters (A) Sagittal views of the substrate binding pocket in the substrate-free inward-facing conformation (PDB: 9LGT). (B) Sagittal views of the substrate binding pocket in the substrate-bounded inward-facing conformation (PDB ID: 8QSR). (C) Structural alignment of substrate-free inward-facing EIIC from Vibrio cholerae (pink, PDB ID: 9LGT) and substrate-bounded inward-facing EIIC from E.coli (blue, PDB ID: 8QSR).

The substrate-binding site is located within the transport domain (TD) of each EIIC protomer and is positioned near the interface with the scaffold domain (SD). This site forms a nearly spherical cavity approximately 9.6 Å in radius and is composed of residues from HP1, HP2b, TM6, and the TM7-α3 loop. These elements collectively define the structural framework required for substrate accommodation ( Figure 4A). The cavity with the narrowest pore diameter (~7.1 Å) is sufficient to accommodate glucose molecules, given the size of glucose (~4.7 Å for the O-1 to O-3 distance). Structural and functional studies of homologous glucose-family EIIC transporters, such as *E*. *coli* PtsG and *Bacillus cereus* BglF, have demonstrated that mutations in conserved residues within HP1, HP2, and the GITEP motif significantly impair sugar binding and phosphorylation activities [ 27, 29] . For example, substitution of the conserved glutamate in the GITEP motif abolishes sugar translocation, whereas perturbations in the HP1/HP2 residues disrupt the alternating access mechanism required for substrate movement. These findings strongly support the functional conservation of these motifs across the Glc family.

Our cryo-EM structure, however, lacks additional electron density within the substrate-binding pocket, indicating a substrate-free conformation. This structure likely represents a state preceding substrate binding or following substrate release. Comparative analysis with inward-facing occluded states from previous studies revealed a larger cavity diameter in our substrate-unbound structure ( Figure 4A,B). This observation aligns with molecular simulations by Roth *et al*. [27],who demonstrated an increase in cavity diameter during glucose release in the inward-facing protomer, suggesting a substrate-free inward-open state. Furthermore, docking studies using HADDOCK, which is based on the substrate-free inward-facing conformation resolved in this study, indicate that glucose molecules are repositioned toward the cytoplasmic side compared with their location in the inward-facing occluded state ( Supplementary Figure S5)[ 26, 27, 30] . This movement suggests a mechanism for substrate release into the cytoplasm, where the substrate is subsequently transferred to the EIIB component of the PTS system.

### Structural insights into the substrate release of the EIIC transporter

The observed cryo-EM structure likely represents the substrate-released state described in the alternating access model of transport [ 31, 32] . According to this model, the transport cycle involves at least two additional conformational states: an outward-open state, which facilitates substrate binding from the periplasm, and an inward-open state, which enables interaction with the EIIB component for substrate phosphorylation and cytoplasmic release. To further elucidate the substrate release mechanism, we compared the substrate-free inward-facing conformation resolved in this study with previously characterized substrate-bound inward-facing structures ( *e*. *g*., PDB: 8QSR). This comparison helps elucidate the final step of the transport cycle, in which substrate release occurs ( Figure 4C).

Typically, the rigid-body motion of the substrate-binding domain is coupled with conformational changes in the N- and C-terminal regions of the amphipathic helix (AH), which is highly conserved across the glucose-specific subfamily of EIIC transporters ( Supplementary Figure S4). Notably, comparison with the substrate-bound inward-facing structure (PDB: 8QSR) revealed no significant global displacement of the AH domain in our substrate-free inward-facing structure. In previous structures, AH, an extension of TM5, forms a continuous α-helix that bends at position P199, aligning parallel to the membrane surface. In contrast, in our structure, the AH retains its overall position but exhibits a discontinuity at position P188 (corresponding to P173 in the substrate-bound structures), resulting in two distinct α-helices. This structural variation likely results from a swinging movement of TM5 away from the substrate-binding center, which could facilitate an upward shift of the substrate-binding domain toward the extracellular side, thereby increasing the available space at the substrate-binding site ( Figure 4C and Supplementary Movie S1).

H226 from HP1b is implicated in the phosphotransfer reaction and is highly conserved within the glucose EIIC superfamily ( Supplementary Figure S4)[ 33, 34] . Further structural comparisons revealed that the HP1a domain, which is structurally connected to AH, rotates and slightly moves toward the substrate-binding center, enabling rotation of the substrate-binding domain. Additionally, the HP1b and β1/β2 regions, in contrast to the previously characterized structure (PDB: 8QSR), swing toward the cytoplasmic side, potentially enlarging the substrate-binding pocket and facilitating glucose release. These observations are consistent with molecular dynamics simulations of the outward-facing bcMalT transporter, which demonstrated significant flexibility in the HP1b region, potentially enabling the substrate to move away from the binding site and facilitating the opening of the substrate-binding pocket [35]. This aligns with our structural findings and further supports the notion that the substrate-binding pocket is dynamically adjustable, playing a key role in the release of the substrate ( Figure 4C and Supplementary Movie S2).

An additional noteworthy feature of our structure is the formation of an intrachain disulfide bond between C240 (in β1) and C254 (in α1) within the same protomer ( Figure 4C). To date, this disulfide bond has not been observed in any currently resolved EIIC structures, suggesting that it may represent a specific interaction. Circular dichroism (CD) revealed an increase in the melting temperature (Tm) for the C240A/C254A double mutant, suggesting that disulfide bonds may fine-tune conformational dynamics rather than static stability ( Supplementary Figure S6). Located at the interface between the scaffold domain (SD) and the transport domain (TD), this disulfide bond likely serves to stabilize the conformation of this region, potentially facilitating the dynamic process of glucose transport.

## Discussion

This study reveals the high-resolution cryo-EM structure of the EIIC transporter from *Vibrio cholerae*, providing a substrate-free, inward-facing conformation critical for understanding the glucose-specific phosphotransferase system (PTS). The substrate-binding pocket, located within the transport domain (TD), has a near-spherical cavity (~9.6 Å radius) capable of accommodating glucose. In the substrate-free state observed in the cryo-EM map, the pocket is enlarged (~7.1 Å pore diameter), which is consistent with a substrate-released state. This configuration likely represents a state poised for glucose translocation into the cytoplasm, where the substrate is subsequently phosphorylated by EIIB.

Structural comparisons with substrate-bound EIIC transporters ( *e*. *g*., *E*. *coli* PDB: 8QSR) reveal dynamic rearrangements in regions such as the amphipathic helix (AH) and helical hairpins (HP1a and HP1b). These domains exhibit rotational and swinging motions that facilitate substrate binding, release, and conformational transitions. This flexibility, which is consistent with previous studies on *B*. *cereus* MalT, underscores the critical role of HP1b in regulating substrate accessibility by modulating the size and orientation of the binding pocket. A unique feature of the EIIC transporter is the intrachain disulfide bond between C240 (in β2) and C254 (in α1), which is located at the SD-TD interface. This disulfide bond, which is absent in previously resolved EIIC structures, likely stabilizes the transporter and may influence its functional dynamics in *Vibrio cholerae*. The structural integrity conferred by this bond represents a novel element for potential therapeutic intervention.

Currently, there appears to be limited direct research on specific inhibitors targeting the EIIC sugar transporter in *Vibrio cholerae*. However, several studies examining the broader phosphoenolpyruvate phosphotransferase system (PTS) and related components provide valuable insights into potential therapeutic strategies. For example, the inhibition of components such as EIIAGlc has been demonstrated to disrupt biofilm formation, a crucial factor for both environmental persistence and pathogenicity [21]. Structural studies, including the current analysis, highlight conformational changes during sugar transport, suggesting that these changes could be exploited in the rational design of EIIC inhibitors [26]. Furthermore, studies on glucose-specific enzyme IIA (EIIAGlc) indicate its dual functionality in regulating metabolism and biofilm formation, revealing potential targets for disrupting the bacterium’s adaptation mechanisms to host environments [36]. While targeted inhibitors against EIIC specifically remain rare, these broader findings underscore promising avenues for future inhibitor development.

The detailed characterization of the EIIC substrate-binding pocket and conformational dynamics lays the groundwork for rational drug design. Targeting the conserved features of EIIC, such as the GITEP motif and key dynamic regions, could disrupt sugar transport, thereby impairing the growth and pathogenicity of *Vibrio cholerae*. Furthermore, the conservation of structural features across glucose-specific EIIC transporters suggests the broader applicability of such therapeutic strategies to pathogens reliant on similar sugar uptake mechanisms.

In conclusion, this study enhances our understanding of the molecular mechanisms governing substrate release and structural transitions in bacterial glucose transporters. The structural insights provided herein establish a foundation for future research into the PTS system and inform the development of targeted antimicrobial therapies to combat cholera and related bacterial infections.

While our study provides structural insights into *Vibrio cholerae* EIIC, the absence of direct functional validation, such as transport assays or substrate-binding studies, precludes definitive linkage of the observed structural features to glucose transport activity. Although biophysical data and comparative analyses with homologs support our hypotheses, mechanistic conclusions about substrate translocation, phosphorylation coupling, or the proposed roles of motifs such as HP1/2 and GITEP remain inferred rather than experimentally confirmed. Furthermore, functional inference about key residues relies heavily on mutagenesis studies in EIIC homologs. While these systems share structural conservation, subtle functional divergences in *Vibrio cholerae* EIIC cannot be excluded, particularly given its unique disulfide bond (C240–C254). Although disrupting this bond increased thermostability in CD experiments, suggesting a role in conformational flexibility, its regulatory function *in vivo* remains to be tested.

## Supporting information

25094FigS1-5-TabS1

## Abbreviations

The cryo-EM map of the EIIC transporter has been deposited in the Electron Microscopy Data Bank (EMDB) under the accession code EMD-63072, and the corresponding model coordinates are available in the Protein Data Bank (PDB) under the accession code 9LGT. All additional data supporting the findings of this study are provided in the main text and Supplementary Data.

## Supplementary Data

Supplementary data is available at *Acta Biochimica et Biophysica Sinica* online.
