# Supplementary material for: Structural insight into Vibrio cholerae EIIC sugar transporter dimer captured in a substrate-free inward-facing state : Vibrio cholerae EIIC dimer in inward-facing state
Source: Acta Biochim Biophys Sin (Shanghai). 2025 Aug 12;58(2):303–10. doi: 10.3724/abbs.2025120 (PMC12900739; doi:10.3724/abbs.2025120)
Supplement: 25094FigS1-5-TabS1 [file 25094FigS1-5-TabS1.docx]

**
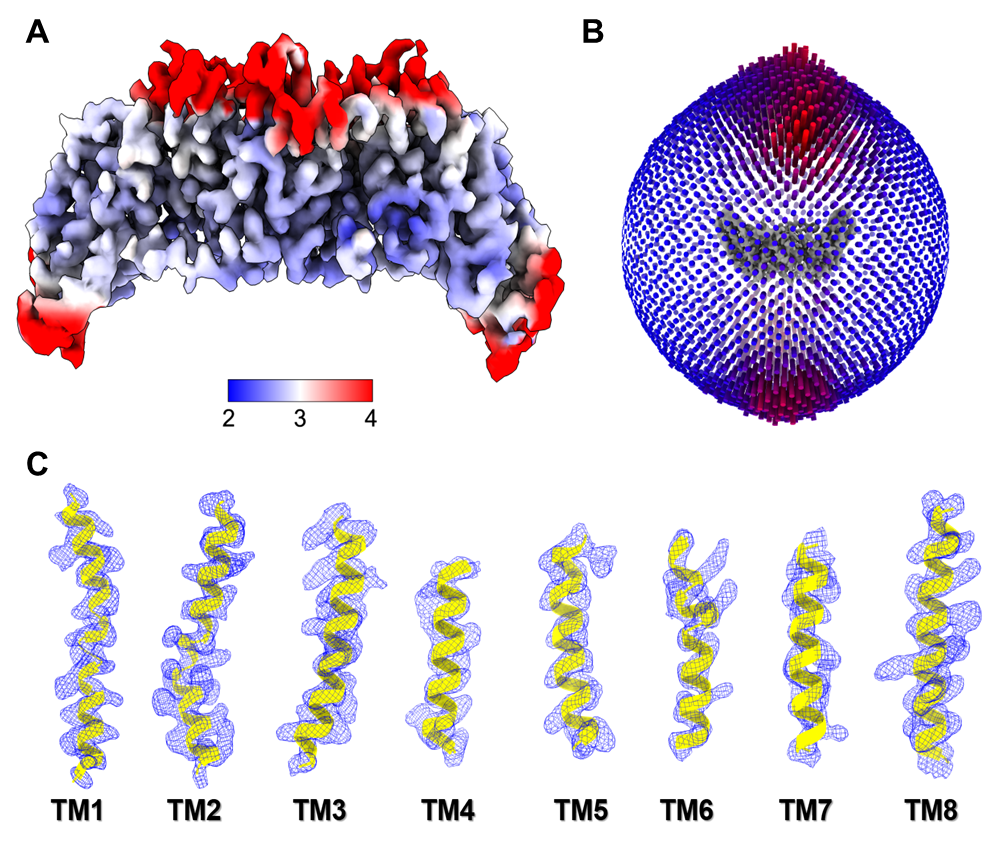
**

**Supplementary Figure S1. Structural resolution and density map of the inward-facing EIIC transporter from *Vibrio cholera*** (A) Local resolution map of the EIIC transporter. (B) Angular distribution plot for the final particle set. (C) Representative cryo-EM density maps of the EIIC transporter.


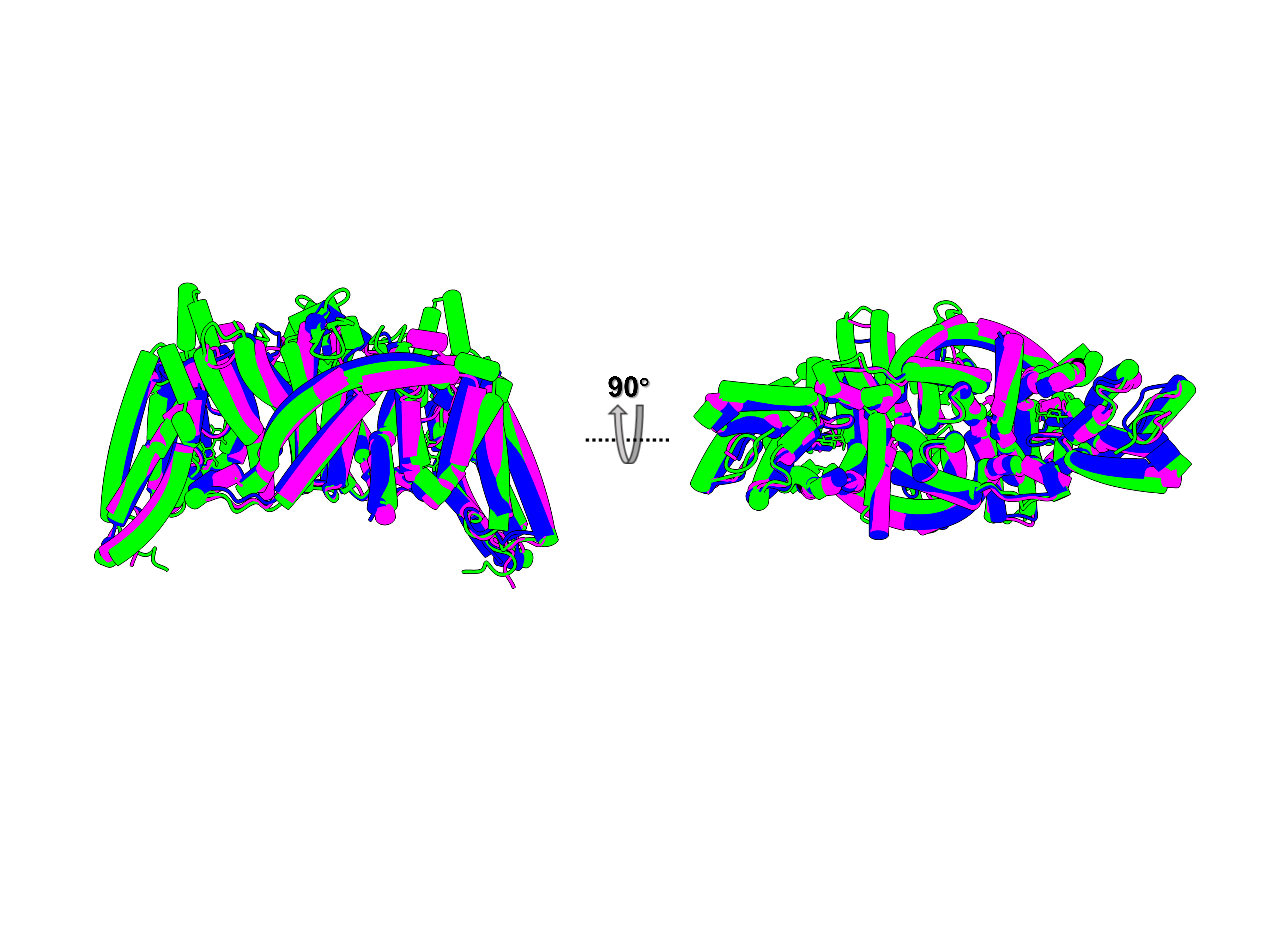


**Supplementary Figure S2. Structural alignment of inward-facing EIIC transporters from different species** Structural alignment of the inward-facing EIIC transporter of MalT from *B. cereus* (green, PDB ID: 6BVG), the substrate-free inward-facing EIIC transporter from *Vibrio cholerae* (pink, PDB ID: 9LGT), and the substrate-bound inward-facing EIIC transporter from *E. coli* (blue, PDB ID: 8QSR).


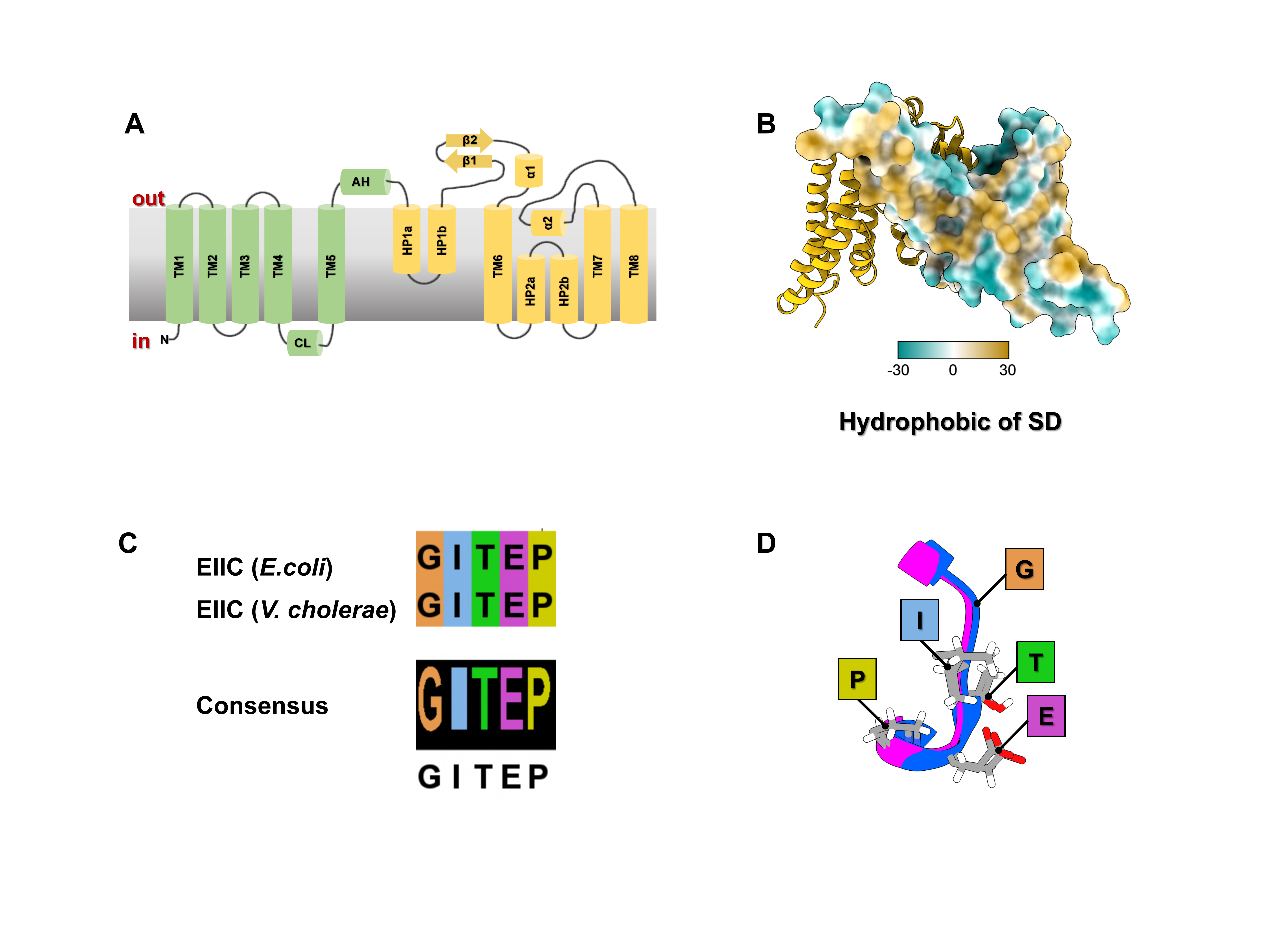


**Supplementary Figure S3. Structural and sequence features of the EIIC transporter** (A) Schematic topology diagram of the EIIC transporter, illustrating the scaffold domain (SD) in green and the transport domain (TD) in yellow. (B) Hydrophobic surface rendering of the SD/TD interface, colored according to the molecular lipophilicity potential (MLP): turquoise for regions with negative MLP and brown for regions with positive MLP. (C) Sequence conservation of the GITEP motif between the EIIC transporter from *Vibrio cholerae* and *E. coli*. (D) Structural conservation of the GITEP motif between the EIIC transporter from *Vibrio cholerae* (pink) and *E. coli* (blue), highlighting the conserved architecture critical for substrate transport and phosphorylation.


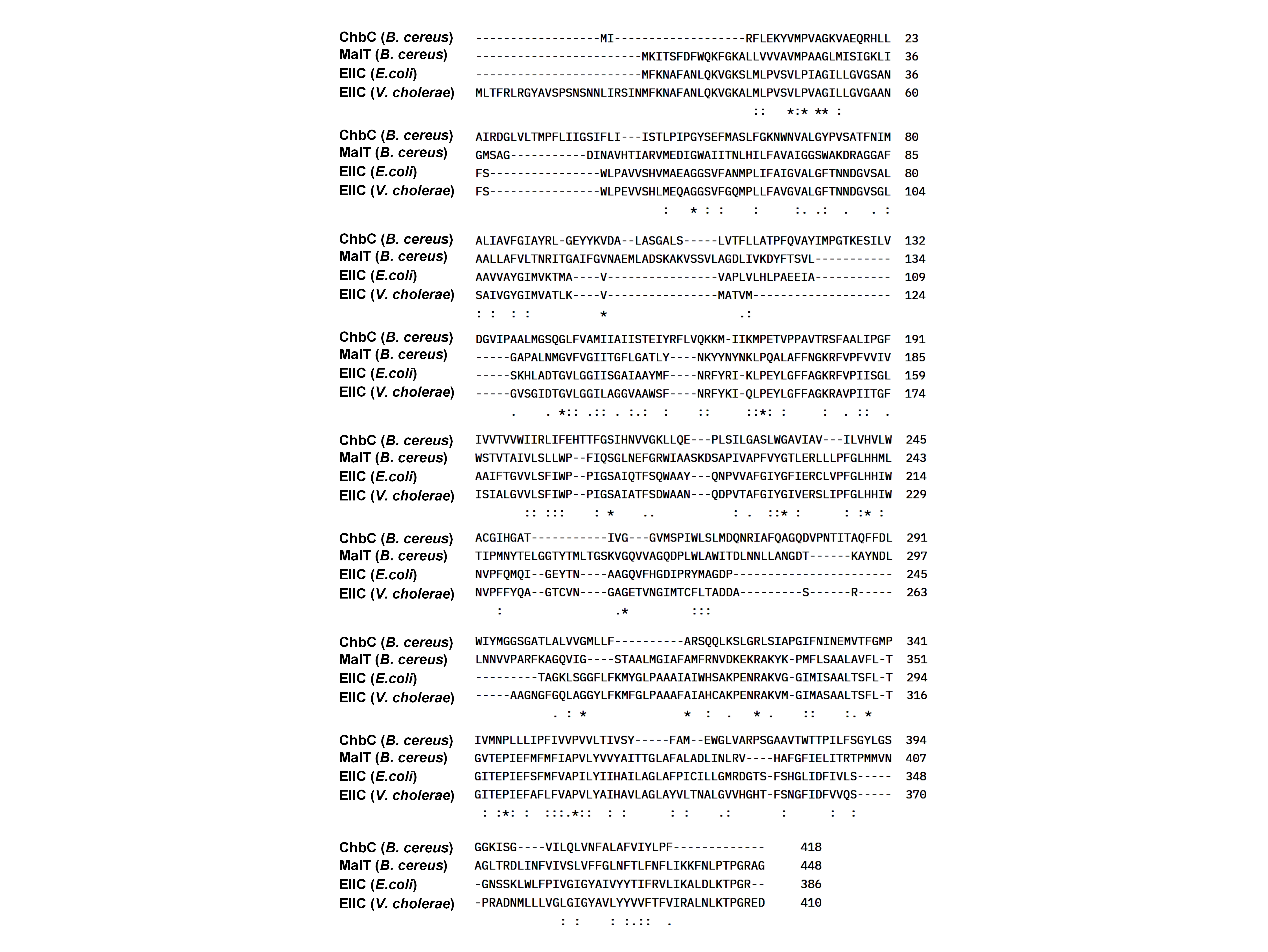


**Supplementary Figure S4.** **Sequence alignment of representative functionally characterized EIIC transporter** All sequences are members of the glucose (Glc) subfamily of the Glucose superfamily, except bcChbC, which is from the lactose (Lac) subfamily. The sequences are: ChbC from *B. cereus* (UniProt Acc # Q72XQ0), MalP from *B. cereus* (UniProt Acc #P54715), MalT from *B. cereus* (UniProt Acc #Q63GK8), EIIC from *E. coli* (UniProt Acc #C3TDU2), and EIIC from *V. cholerae* (UniProt Acc #C3LNW4).


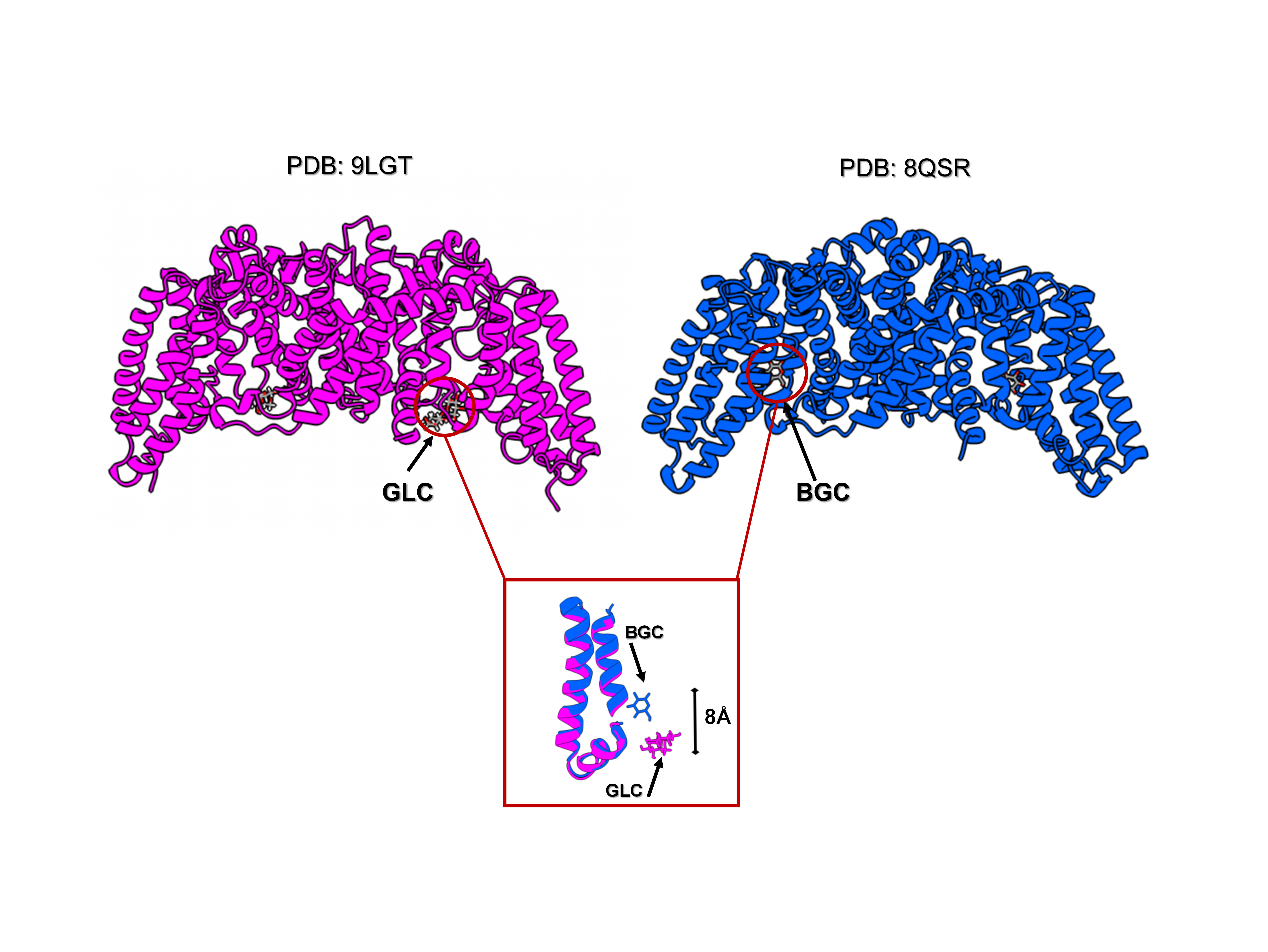


**Supplementary Figure S5.** **Substrate positional changes in inward-facing EIIC transporters** Structural alignment of glucose-docked inward-facing EIIC from *Vibrio cholerae* (pink, PDB ID: 9LGT) and β-D-glucopyranose (BGC)-bound inward-facing EIIC from Escherichia coli (blue, PDB ID: 8QSR). The comparison reveals conformational differences in the substrate position within the binding pocket, highlighting structural transitions associated with substrate binding and release. Key shifts in the substrate location reflect the dynamic rearrangements required for efficient sugar transport in the phosphotransferase system.


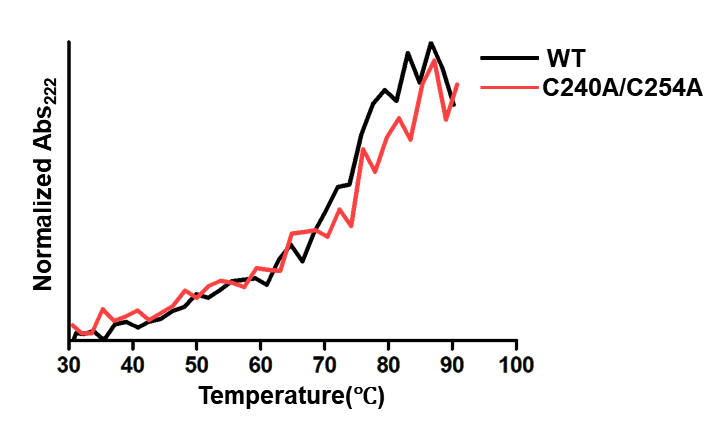


**Supplementary Figure S6. Thermodynamic stability by circular dichroism** The thermal unfolding curves were recorded at a wavelength of 220 nm to monitor the changes of transmembrane helices.

**Supplementary Table S1. Cryo-EM data collection, refinement and validation statistics**

| Parameter | Data |
| --- | --- |
| Data collection and processing | |
| EM equipment | 300 |
| Voltage (kV) | Gatan K3 |
| Detector | 0.668 |
| Pixel size (Å) | −1.5 ~ 2.5 |
| Defocus range (µm) | 130,000 × |
| Magnification | 32 |
| Frames | 50 |
| Total dose (*e^−^*/Å^2^) | 5864 |
| Number of collected micrographs | 3200 |
| Number of selected micrographs | 87,760 |
| Number of used particles | 3.1 |
| Map Resolution (Å) | C2 |
| Symmetry | 300 |
| Refinement | |
| Initial model | AlphaFold |
| Model Resolution (Å) | 3.6 |
| Model composition | |
| Chains | 2 |
| Non-hydrogen | 2825 |
| Residues | 764 |
| B factors (Å^2^) | |
| Protein | 36.58/84.26/52.23* |
| Ligand | *−*/*−*/*−* |
| Water | *−*/*−*/*−* |
| R.m.s. deviations | |
| Bonds (RMSD) | 0.005 |
| Bonds length (Å) | 0.665 |
| Validation | |
| MolProbity | 1.65 |
| Clashscore | 7.0 |
| Ramachandran plot statistics (%) | |
| Preferred | 96.0 |
| Allowed | 4.0 |
| Outlier | 0.00 |

*Minimum/maximum/mean
